# Synthesis of [4*S*‐^2^H]NADH, [4*R*‐^2^H]NADH, [4‐^2^H_2_]NADH and [4‐^2^H]NAD^+^ cofactors through heterogeneous biocatalysis in heavy water

**DOI:** 10.1002/jlcr.3899

**Published:** 2021-01-26

**Authors:** Jack S. Rowbotham, Adam P. Hardy, Holly A. Reeve, Kylie A. Vincent

**Affiliations:** ^1^ Department of Chemistry, Inorganic Chemistry Laboratory University of Oxford Oxford UK

**Keywords:** D_2_O, ^2^H_2_O, deuterated cofactor, dihydrogen gas (H_2_), immobilised enzymes

## Abstract

This practitioner protocol describes the synthesis of a family of deuterated nicotinamide cofactors: [4*S*‐^2^H]NADH, [4*R*‐^2^H]NADH, [4‐^2^H_2_]NADH and [4‐^2^H]NAD^+^. The application of a recently developed H_2_‐driven heterogeneous biocatalyst enables the cofactors to be prepared with high (>90%) ^2^H‐incorporation with ^2^H_2_O as the only isotope source.

## Graphical abstract

This protocol describes the use of a H_2_‐driven heterogeneous biocatalyst to prepare a suite of deuterated nicotinamide cofactors: [4*S*‐^2^H]NADH, [4*R*‐^2^H]NADH, [4‐^2^H_2_]NADH, and [4‐^2^H]NAD^+^. The methodology requires only heavy water (^2^H_2_O and D_2_O) as the source of deuterium isotopes.

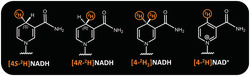

## 1 INTRODUCTION

**FIGURE 1:**
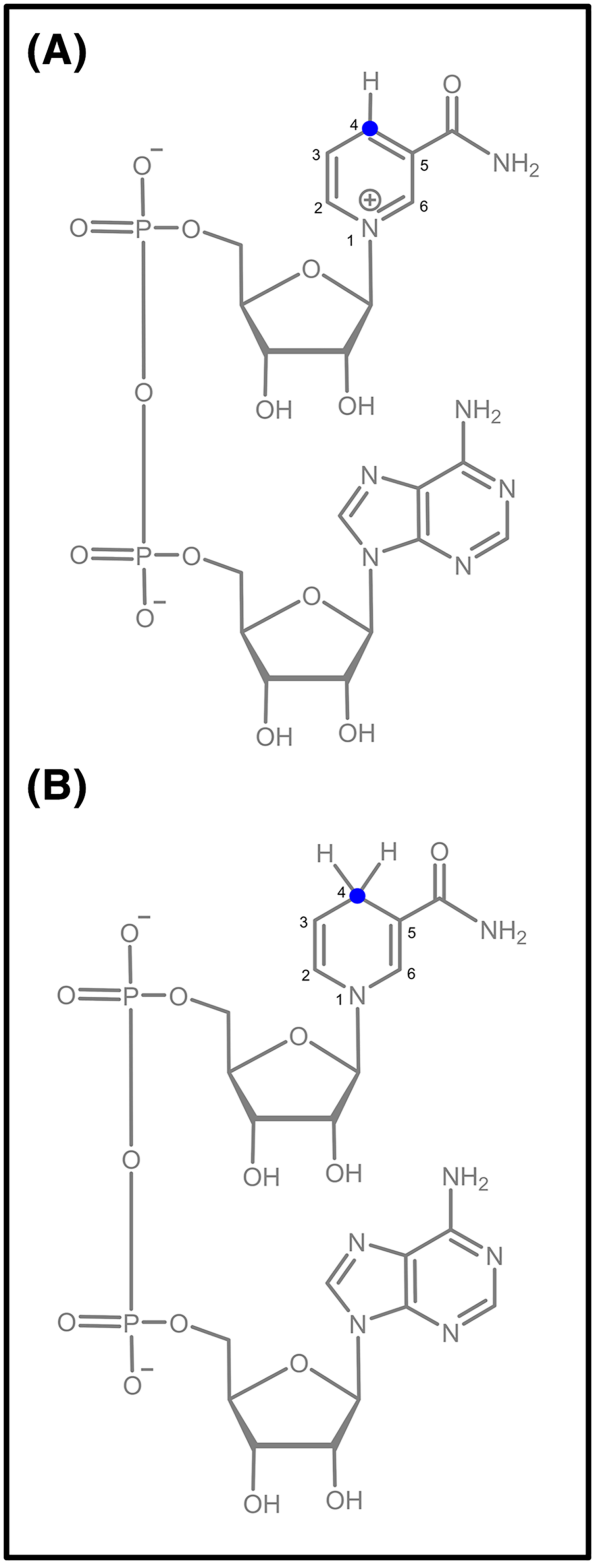
Structures of nictotinamide cofactors (A) NAD^+^ and (B) NADH. The target site for deuteration (the 4‐position of nicotinamide ring) is highlighted in blue

Nicotinamide cofactors in their oxidised (NAD^+^) and reduced (NADH) forms (Figure 1) mediate electron transfer in a wide range of redox enzyme (oxidoreductase)‐catalysed reactions. Isotopic labelling of these molecules is a well‐established technique for probing enzymatic mechanisms,^1^ exemplified by the work of JW Cornforth on the stereoselectivity of biocatalysed processes, for which he was awarded the 1975 Nobel Prize in Chemistry.^2^, ^3^ More recently, deuterated NADH has been shown to be a useful tool for the preparation of synthetically challenging asymmetric heavy drug analogues.^4^

Deuterated and tritiated forms of NADH may be prepared by reducing NAD^+^ in ^2^H_2_O (D_2_O)/^3^H_2_O (T_2_O) with sodium dithionite,^2^ or in ^1^H_2_O with a deuteride/tritide salt.^5^ Although simple, these methods lack selectivity as to which face of the nicotinamide ring the ^2^H/^3^H atom is added. Alternatively, an oxidoreductase of the desired selectivity may be supplied with NAD^+^ and a ^2^H‐ or ^3^H‐labelled substrate.^6^ For example, Kohen et al. have demonstrated the preparation and isolation of a range of ^2^H‐labelled nicotinamide cofactors (amongst other isotopologues) through the reaction of NAD^+^ with a glucose dehydrogenase and D‐[1‐^2^H]glucose or an alcohol dehydrogenase (ADH) with [2‐^2^H]isopropanol.^7^, ^8^, ^9^, ^10^, ^11^ Despite the elegance of these methods, they still require a ^2^H‐ or ^3^H‐labelled organic precursor, which reduces the atom economy of the process, and may add complexity in, for example, the separation of the product.

We have recently reported a route to deuterated NADH that uses H_2_ as the reducing agent and ^2^H_2_O as the source of deuterium isotopes.^4^ This method utilises a heterogeneous biocatalyst, whereby a hydrogenase (capable of oxidising H_2_) is co‐immobilised with an NAD^+^ reductase (capable of reducing NAD^+^) on an electronically conducting carbon support. Separation of the two enzymatic sites allows for high levels of deuterium incorporation into the cofactor, but avoids the use of a labelled reductant. The selectivity of the NAD^+^ reductase also ensures that only [4*S*‐^2^H]NADH is formed.

In this practitioner protocol, we describe how, by extension of the heterogeneous biocatalytic method described above, [4*R*‐^2^H]NADH, [4‐^2^H_2_]NADH and [4‐^2^H]NAD^+^ may all be prepared, in addition to [4*S*‐^2^H]NADH. All of the enzymes used can be purchased from named suppliers or prepared by established molecular biology techniques. Alternatively, reasonable requests for material (catalysts or deuterated cofactors) can be made through the corresponding authors.

### 1.1 Synthetic approach

**FIGURE 2:**
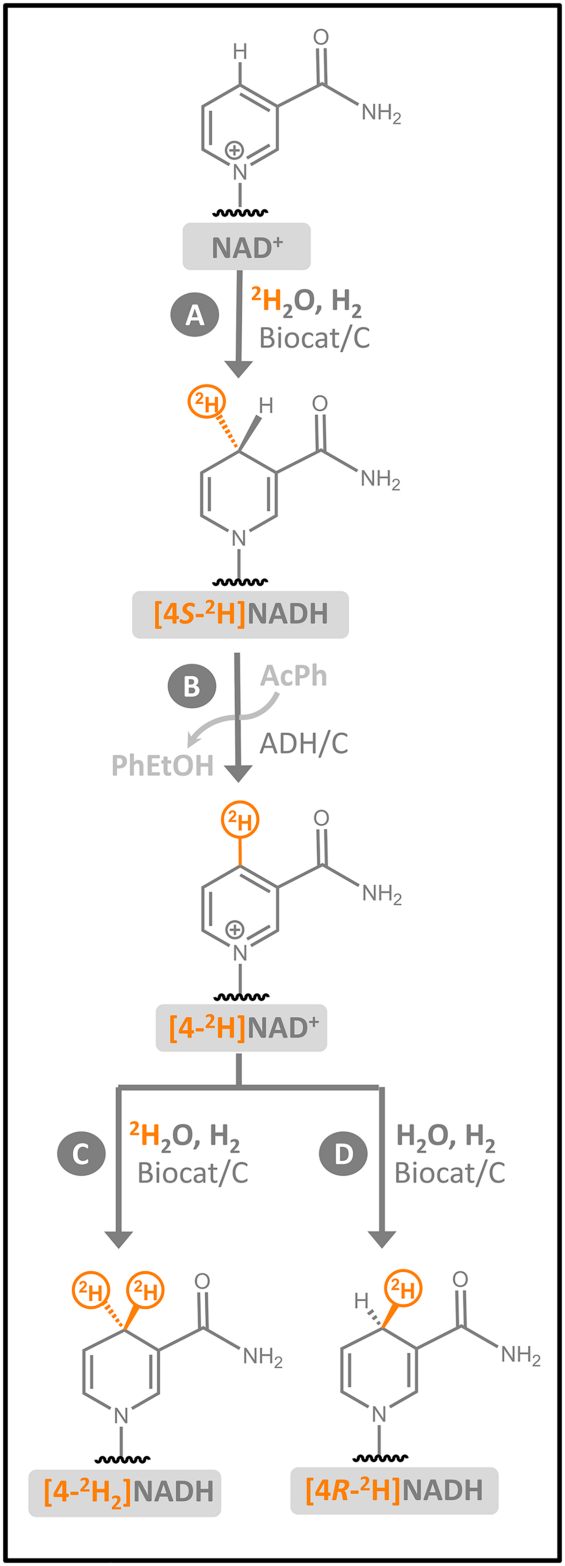
Overview of synthetic approach used to prepare [4*S*‐^2^H]NADH (Step A), [4‐^2^H]NAD^+^ (Step B), [4‐^2^H_2_]NADH (Step C) and [4*R*‐^2^H]NADH (Step D)

The approach shown in Figure 2 was used to prepare different isotopologues of NAD^+^ and NADH. In brief, [4*S*‐^2^H]NADH was first prepared from NAD^+^ by using the H_2_‐driven heterogeneous biocatalyst (Biocat/C) operating in ^2^H_2_O (Step A). This [4*S*‐^2^H]NADH was then re‐oxidised by reaction with excess acetophenone (AcPh) and a commercial ADH immobilised on carbon (ADH/C). The ADH was chosen such that it selectively removed ^1^H to leave [4‐^2^H]NAD^+^ (Step B).

Following the removal of the ADH/C, unreacted AcPh and phenylethanol (PhEtOH) side‐product, the [4‐^2^H]NAD^+^ was either left in ^2^H_2_O or dried and transferred into ^1^H_2_O. Reduction of [4‐^2^H]NAD^+^ using the H_2_‐driven heterogeneous biocatalyst yielded the cofactors [4‐^2^H_2_]NADH (when performed in ^2^H_2_O, Step C) and [4*R*‐^2^H]NADH (when performed in ^1^H_2_O, Step D). In all cases, the removal of the catalyst was easily achieved by centrifugation, owing to the carbon support employed to heterogenise the catalysts.

In the work reported here, the crude reaction mixtures were used directly after each step, and no purified or isolated cofactors were prepared at any stage. However, if further purification or isolation was required (particularly to remove buffer salts, unreacted starting material or impurities arising from cofactor degradation), we refer the reader to suitable protocols reported in this journal^8^ and elsewhere.^12^

## 2 EXPERIMENTAL DETAILS

### 2.1 General reagents and conditions

All commercial reagents were used as received and without further purification, unless specified. NAD^+^ and NADH were purchased from Prozomix * and all other reagents from Sigma Aldrich. † Deionised MilliQ water (Millipore, 18 MΩ cm) was used for nondeuterated solutions, and ^2^H_2_O (99.98%, Sigma Aldrich) was used for deuterated solutions. When required, Trizma base was exchanged into ^2^H_2_O prior to use to minimise ^1^H contamination and then adjusted to the required p^2^H (pD) with ^2^HCl. All aqueous solutions were sparged with dry N_2_ for 60 min prior to use in order to deoxygenate them.

The heterogeneous biocatalyst (Biocat/C) comprised a NiFe‐hydrogenase (Hydrogenase 1 from *Escherichia coli*) and an NAD^+^ reductase (I64A variant of the soluble hydrogenase from *Ralstonia eutropha*) co‐immobilised on carbon black particles (Black Pearls 2000 [BP2000], Cabot Corporation) ‡ according to published protocols.^4^ In a typical experiment, 400 μg of carbon (supporting around 80‐140 μg each of hydrogenase and NAD^+^ reductase) was used per 1 ml of reaction mixture. The commercial ADH (ADH105, Johnson Matthey) § was immobilised on carbon particles by suspending 50 μl of concentrated solution (20 mg/ml) to an equal volume of sonicated BP2000 particles (20 mg/ml) for 30 min at 4°C. Following centrifugation (13,800 × *g*, 5 min), the supernatant was removed, and the particles were washed once with 100‐μl ^2^H_2_O.

**FIGURE 3:**
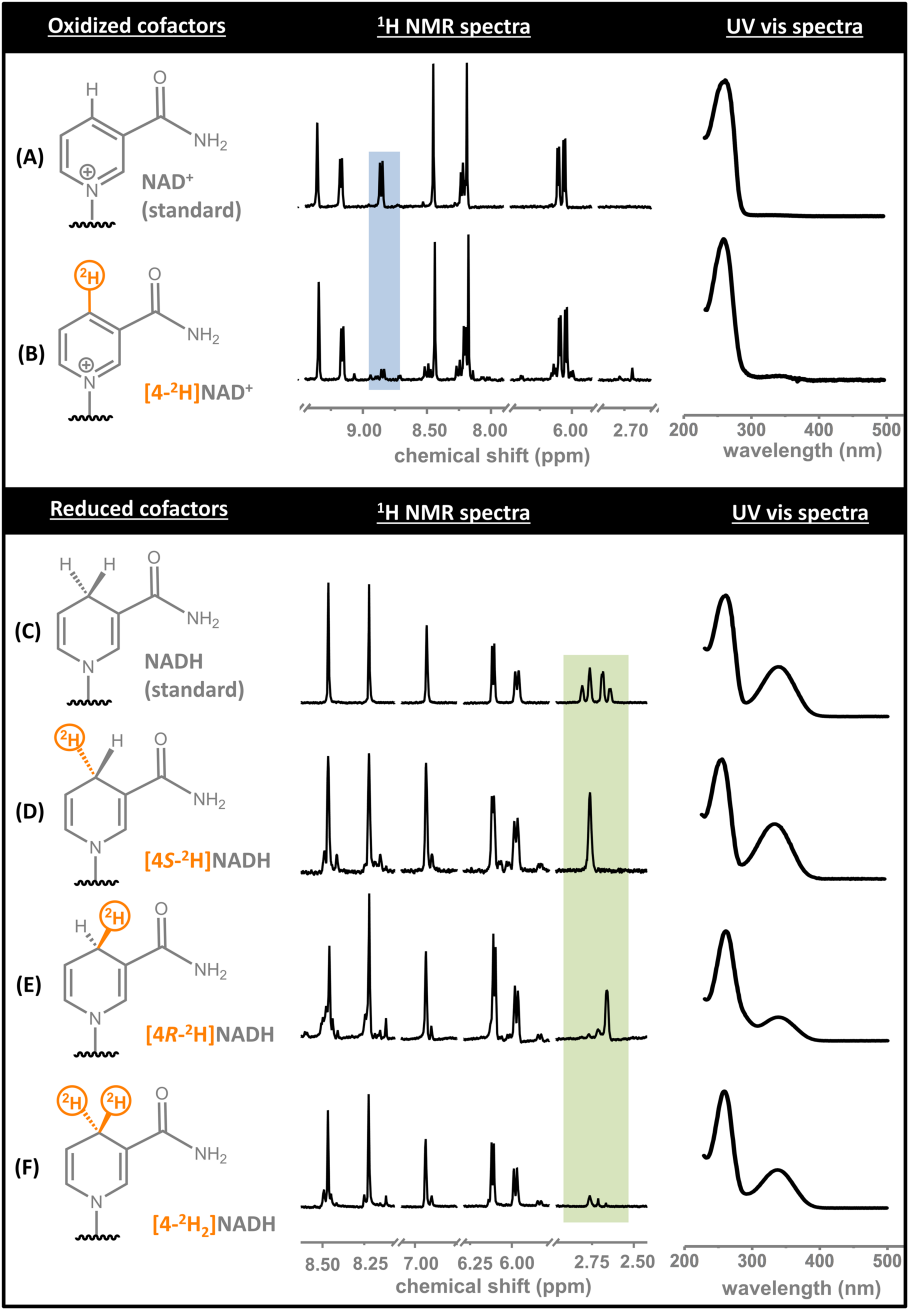
Diagnostic regions of ^1^H nuclear magnetic resonance (NMR) spectra (400 MHz, H_2_O/^2^H_2_O, pH 8.0) and UV‐vis spectroscopy (H_2_O, pH 8.0) for different isotopologues of oxidised (A,B) and reduced (C–F) nicotinamide cofactors: (A) NAD^+^ (commercial standard), (B) [4‐^2^H]NAD^+^, (C) NADH (commercial standard), (D) [4*S*‐^2^H]NADH, (E) [4*R*‐^2^H]NADH and (F) [4‐^2^H_2_]NADH. The highlighted regions show signals arising from the 4‐position of the nictotinamide rings for the oxidised (blue) and reduced (green) cofactors

The reactions reported here were set up in a glovebox with an N_2_ atmosphere (<0.1‐ppm O_2_), and those with pressurised H_2_ were conducted in a Büchi Tinyclave. ¶ It has been demonstrated elsewhere that a rigorously anaerobic setup is not required, and that conventional benchtop synthetic apparatus (N_2_‐flushed round‐bottom flask, H_2_ balloon) can be used as an alternative.^4^ Products were analysed using UV‐vis and ^1^H nuclear magnetic resonance (NMR) (400 MHz) spectroscopies (Figure 3), according to standard procedures.^13^

#### 2.1.1 Step A: Synthesis of [4*S*‐^2^H]NADH from [4‐^1^H]NAD^+^

An aliquot (100 μg) of Biocat/C (prepared as above) was added to 500 μl of [^2^H_5_]‐Tris.^2^HCl (100 mM, p^2^H 8.0) containing 4‐mM NAD^+^, which had been presaturated with ^1^H_2_ gas. The reaction solution was sealed under 2‐bar H_2_ for 16 h, whilst rocking at 30 rpm. Conversion of NAD^+^ to [4*S*‐^2^H]NADH was 95%, with >98% incorporation of ^2^H. No other products were detected by ^1^H NMR spectroscopy.

#### 2.1.2 Step B: Synthesis of [4‐^2^H]NAD^+^ from [4*S*‐^2^H]NADH

To a deoxygenated solution of [4*S*‐^2^H]NADH (1 ml, 4 mM) in [^2^H_5_]‐Tris.^2^HCl (100 mM, p^2^H 8.0), ADH/C particles (2,000 μg) and AcPh (10 mM) were added. The whole solution was shaken under N_2_ for a period of 5 days. The ADH/C particles were removed by centrifugation (13,800 × *g*, 5 min), and the remaining acetophenone and formed phenylethanol were removed by extraction with CHCl_3_ (3 × 500 μl). A conversion of 95% to the [4‐^2^H]NAD^+^ form was observed, with full retention of ^2^H at the 4‐position of the nicotinamide ring from the starting material. It was possible to conduct Step B in a shorter time using the ADH in solution (rather than immobilised), though this required subsequent precipitation of the enzyme by addition of acetonitrile (1 ml) and removal by centrifugation (13,800 × *g*, 5 min).

#### 2.1.3 Step C: Synthesis of [4‐^2^H_2_]NADH from [4‐^2^H]NAD^+^

Following on from Step B, Biocat/C were added at a loading of 200 μg ml^−1^ to a solution of [4‐^2^H]NAD^+^ (500 μl, 3 mM) in [^2^H_5_]‐Tris.^2^HCl (100 mM, p^2^H 8.0) that had been presaturated with ^1^H_2_ gas. The mixture was then sealed under 2‐bar H_2_ and rocked at 30 rpm for 16 h. Conversion to the desired [4‐^2^H_2_]NADH was >90%, with 95% ^2^H incorporation at the 4‐position of the reduced nicotinamide ring.

#### 2.1.4 Step D: Synthesis of [4*R*‐^2^H]NADH from [4‐^2^H]NAD^+^

A solution of [4‐^2^H]NAD^+^ (1 ml, 3 mM) in [^2^H_5_]‐Tris.^2^HCl (100 mM, p^2^H 8.0) was prepared according to Step B, and an equal volume of acetonitrile was added to facilitate precipitation during the drying stage. The solution was gently evaporated to dryness under reduced pressure on a rotary evaporator (at 20°C), and the solid was redissolved in deionised ^1^H_2_O. The evaporation and redissolution steps were repeated twice more to fully exchange all of the remaining ^2^H to ^1^H. The solution was evaporated a final time to leave an off‐white solid consisting of cofactor and buffer salts. The solid was transferred to a glovebox and redissolved in deoxygenated deionised ^1^H_2_O (1 ml). The cofactor solution was subsequently saturated with ^1^H_2_ gas. An aliquot (200 μg) of Biocat/C catalyst was added to the solution, which was subsequently sealed under 2‐bar H_2_ and shaken at 30 rpm for 18 h. The Biocat/C catalyst was removed by centrifugation (13,800 × *g*, 5 min). Subsequent analysis indicated around 50% conversion to the reduced cofactor, with a high selectivity (>90%) for the [4*R*‐^2^H]NADH isotopologue. Some degradation of the cofactor was observed during the evaporation steps, and separation by ion exchange chromatography would be needed to obtain a pure sample in this case.^8^, ^12^

### 2.2 Product analysis

UV‐vis and ^1^H NMR (400 MHz) spectra arising from the analysis of the products in Steps A–D are shown in Figure 3 alongside commercial samples of NAD^+^ and NADH. The ratio of oxidised to reduced cofactor in the samples was determined by comparing the ratio of the absorption at 340 nm with that at 260 nm in the UV‐vis spectra.^14^ Analysis of the isotopic composition of the cofactors was achieved by analysing diagnostic peaks in the ^1^H NMR spectra. In the case of the oxidised cofactors, the doublet at δ 8.80 ppm is assigned to the proton at the 4‐position of the nicotinamide ring. As expected, the signal seen in the spectrum of NAD^+^ (Figure 3A) is almost absent for [4‐^2^H]NAD^+^ (Figure 3B). In the case of the reduced cofactors, the diagnostic region for the protons on the 4‐position of the nicotinamide ring is between δ 2.60 and δ 2.85 ppm.^15^ In the unlabelled commercial NADH sample (Figure 3C), the two heavily roofed doublets at δ 2.80 and 2.68 ppm correspond to the pro‐*R* and pro‐*S* protons, respectively. Upon single deuteration at the 4‐position to give either [4*S*‐^2^H]NADH (Figure 3D) or [4*R*‐^2^H]NADH (Figure 3E), the signals collapse into *pseudo‐*singlets at δ 2.77 and δ 2.67 ppm, respectively. Double deuteration at the same site to give [4‐^2^H_2_]NADH leads to almost the complete disappearance of peaks in this region, as expected (Figure 3F).

## 3 CONCLUSIONS AND OUTLOOK

This practitioner protocol describes the synthesis of four important nicotinamide cofactor isotopologues: [4*S*‐^2^H]NADH, [4*R*‐^2^H]NADH, [4‐^2^H_2_]NADH and [4‐^2^H]NAD^+^. The main advantages over previous synthetic methods arise from the atom economy of using H_2_ gas as a reductant and the simplicity of using ^2^H_2_O as an isotope source, avoiding the requirement for carbon‐based co‐reagents. Furthermore, the inherent heterogeneous nature of the H_2_‐driven biocatalyst makes reaction work‐up straightforward, and similar strategies have been demonstrated for translation into continuous flow.^16^ Whilst the work presented here concerns cofactor deuteration, preliminary experiments in ^3^H_2_O (37 MBq g^−1^) # indicate that the same procedures should be translatable for similar tritiation.

## CONFLICT OF INTEREST

A patent application detailing some of this research was filed through Oxford University Innovation (February 2018).

## Footnotes

* Prozomix Ltd., Building 4, West End Industrial Estate, Haltwhistle, Northumberland, NE49 9HA, UK.

† Sigma Aldrich, The Old Brickyard, New Road, Gillingham, Dorset, SP8 4XT, UK.

‡ Cabot Corporation, 4400 North Point Parkway, Suite 200, Alpharetta, GA 30022, USA.

§ Johnson Matthey Catalysis and Chiral Technologies, Orchard Road, Royston, Hertfordshire, SG8 5HE, UK.

¶ Ken Kimble Reactor Vessels Ltd., 85 Thomas Way, Lakesview International Business Park, Hersden, Canterbury, Kent, CT3 4NH, UK.

# PerkinElmer LAS (UK) Ltd., Chalfont Road, Seer Green, Beaconsfield, Buckinghamshire, HP9 2FX, UK.
